# The effectiveness of a health promotion intervention on the meaning of life, positive beliefs, and well-being among undergraduate nursing students: One-group experimental study

**DOI:** 10.1097/MD.0000000000019470

**Published:** 2020-03-06

**Authors:** Fu-Ju Tsai, Yih-Jin Hu, Gwo-Liang Yeh, Cheng-Yu Chen, Chie-Chien Tseng, Si-Chi Chen

**Affiliations:** a Department of Nursing, Fooyin University, Kaohsiung; b Department of Health Promotion and Health Education, National Taiwan Normal University; c Department of Education, National Taipei University of Education, Taipei, Taiwan, ROC.

**Keywords:** health promotion, meaning of life, positive beliefs, undergraduate nursing students, well-being

## Abstract

Nursing educators have a responsibility to value undergraduate nursing students’ physical, psychological, spiritual, and social health promotion.

The purpose of the study was to examine the effectiveness of a health promotion intervention concerning meaning of life, positive beliefs, and well-being among undergraduate nursing students in a health promotion curriculum.

The study was adopted a pretest, posttest, and post post-test design in 1-group experimental study with a purposive sample of 112 undergraduate nursing students who attended in a health promotion curriculum and voluntarily completed a reliable 3-part questionnaire (content validity index = 0.95; Cronbach's αs = meaning of life, 0.97; positive beliefs, 0.94; and well-being 0.96).

Undergraduate nursing students showed significant (all *P* < .001) improvements on the meaning of life, positive beliefs, and well-being immediately after the intervention, which were sustained over time.

Nursing educators should incorporate these variables into the health promotion curriculum to enhance undergraduate nursing students’ physical, psychological, spiritual, and social health promotion.

## 1 Introduction

In 1948, World Health Organization (WHO) focused on physical, psychological, social health; 1998 WHO comprised overall health development regarding physical, psychological, spiritual, and social aspects of health promotion.^[1]^ Health is a central concept in nursing education. Specifically, health promotion is critical for undergraduate nursing students; therefore, nursing school curriculum development should include health literacy for health promotion and physical education.^[2]^ Positive and negative health concepts are included in nursing education to develop undergraduate nursing students’ health promotion skills.^[3,4]^ Currently, web-based health information is used to develop undergraduate nursing students’ skills in caring for patients’ health and safety.^[5]^ Nursing educators should promote undergraduate nursing students’ physical, psychological, spiritual, social health to ensure they have meaning of life, positive beliefs, and well-being.^[6]^

First, meaning of life comprises traits such as love, kindness, honesty, hope, gratitude, and social intelligence.^[7]^ It also includes physical activity, good eating habits, and social support, which are associated with physical, mental, and social health promotion.^[8]^ Meaning of life also acts a protective factor against depression, hopelessness, and suicidal ideation,^[9]^ including mediating the influence of mental health on suicidal ideation.^[10]^ Further, spiritual health is associated with life orientation and psychometric properties,^[11]^ and psychosocial health is associated with well-being including religious meaning, life meaning, life satisfaction, spirituality, social support, and quality of life.^[12]^ Meaning of life also increases one's sense of life fulfillment for dying patients to have social support, life satisfaction, and quality of life.^[13]^ In sum, a person's meaning of life is associated with their psychological well-being and mental health;^[14]^ therefore, people need to understand health promotion and establish goals for lifestyle change.^[15]^

Second, positive beliefs are an essential element to manage and face adaptation to individual errors in the learning process.^[16]^ People have positive beliefs that are associated with disease-treatment outcomes.^[17]^ Positive beliefs can increase personal values and mental health, which help offset negative emotions.^[18]^ Positive beliefs can also aid self-perceived health and life satisfaction among rehabilitation students.^[19]^ Optimism is also considered critical to successful aging.^[20]^

Third, well-being is associated with life satisfaction, empowerment, positivity, humor, loneliness, and emotional self-efficacy.^[21]^ Both depression and psychological well-being are associated with physical health outcomes.^[22]^ Further, promoting undergraduate nursing students’ emotional well-being is positively related to their social health and self-concept in the classroom.^[23]^

Diverse health promotion interventions employing distinct teaching methods can promote undergraduate nursing students’ physical, psychological, spiritual, and social health promotion. For example, simulation learning increases transfer of learning and take a role on the behavioral practice in daily life.^[24]^ After several-week intervention, a smoking cessation intervention among undergraduate nursing students displayed significant results in the smoking cessation with health promotion.^[25]^ In sum, nursing educators have a responsibility to utilize diverse teaching methods to help undergraduate nursing students develop their meaning of life,^[26]^ positive beliefs,^[27]^ and well-being,^[28]^ and to enhance their physical, psychological, spiritual, and social health promotion.

### 1.1 Purpose

The purpose of study was to explore the effectiveness of health promotion intervention among undergraduate nursing students on the meaning of life, positive beliefs, and well-being regarding physical, psychological, spiritual, and social health promotion in a health promotion curriculum.

## 2 Methods

### 2.1 Framework

**Figure 1:**
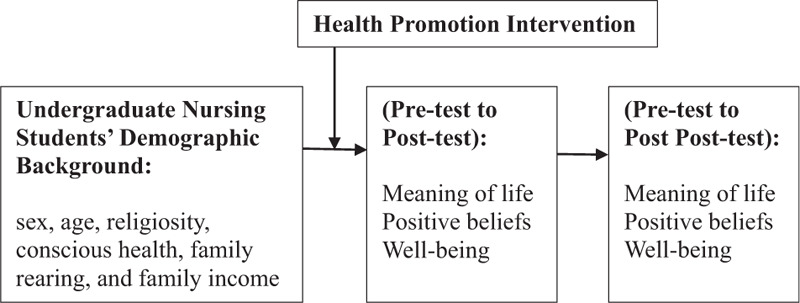
This framework of the study.

This framework of the study was as follows. Undergraduate nursing students’ background included sex, age, religiosity, conscious health, family rearing, and family income. After undergraduate nursing students completed the health promotion intervention, they were examined the immediate effects (pretest to posttest immediately after intervention) and delayed effects (pretest to post posttest after intervention 5 weeks) (see Fig. 1).

### 2.2 Design

This study was adopted a pretest, posttest, and post posttest design in 1-group experimental study.

### 2.3 Participants and setting

A purposive sample was used in this study. All 120 participants were undergraduate nursing students who attended in a health promotion curriculum. Overall, 112 undergraduate nursing students voluntarily completed the pretest, posttest, and post post-test of the questionnaires concerning meaning of life, positive beliefs, and well-being in a health promotion curriculum.

### 2.4 Health promotion intervention

In the health promotion curriculum, which lasted 3 weeks, a nursing educator provided 6 hours of lecture addressing physical, psychological, spiritual, and social health promotion with the aim of increasing undergraduate nursing students’ meaning of life, positive beliefs, and well-being (see Table 1). PowerPoint, YouTube, e-books, and Internet movies were all utilized in the health promotion intervention for providing undergraduate nursing students.

**Table 1:**
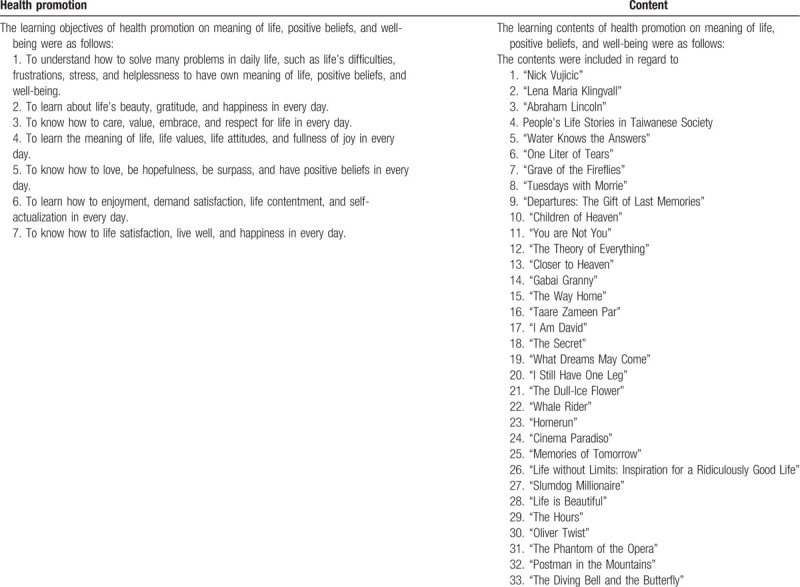
Health promotion intervention.

### 2.5 Ethical considerations

This 1-group experimental study was authorized by the Institutional Review Board of Yuan's General Hospital (IRB No. 20171130C) in Taiwan, ROC. Undergraduate nursing students decided to do the questionnaires on the pretest, posttest, and post posttest. A total of 120 (100.00%) undergraduate nursing students participated in 1-group experimental study in a health promotion curriculum. Finally, 112 (93.33%) undergraduate nursing students voluntarily finished the questionnaires on the 3 times for collecting pretest, posttest, and post posttest.

### 2.6 Measures

A 56-item questionnaire of the measures was followed by the authors, Ho,^[29]^ Lin and Yu^[30]^ on the scales of life attitude profile, positive coping, spirituality, and well-being. The questionnaire was used to explore undergraduate nursing students’ learning effectiveness concerning their meaning of life (25 items; seek meaning, 1–9; life purpose, 10–13; life control, 14–20; and accept misery, 21–25), positive beliefs (11 items), and well-being (20 items; personal well-being, 1–5; community well-being, 6–10; environmental well-being, 11–15; and transcendental well-being, 16–20). The questionnaire also included all information about undergraduate nursing students’ sex, age, religiosity, conscious health, family rearing, and family income. A 5-point Likert scale ranging from completely disagree (1 point) to completely agree (5 points) was used. The content validity index of the study questionnaire was 0.95, as established by 7 expert scholars. After a pilot test (n = 31), the reliabilities of the 3-part measurement were as follows: meaning of life, Cronbach's α = 0.97; positive beliefs, Cronbach's α = 0.94; and well-being, Cronbach's α = 0.96.

### 2.7 Data collection and analyses

A researcher administered the pretest, posttest, and post posttest questionnaires to undergraduate nursing students and explained that the questionnaires would be used to explore undergraduate nursing students’ meaning of life, positive beliefs, and well-being after the health promotion intervention in a health promotion curriculum. Undergraduate nursing students in all 3-part questionnaires were voluntary in the 1-group experimental study (response rate = 93.33%). Questionnaires were collected from February 26, 2018, to May 31, 2018. One hundred twelve undergraduate nursing students completed all 3-part questionnaires for data collection in the study.

SPSS 23.0 was used to analyze all study data. Frequencies; percentages; pretest, posttest, and post posttest means and standard deviations; paired *t* tests; and *P* values were calculated in the 1-group experimental study.

## 3 Results

### 3.1 Undergraduate nursing students’ demographic background

Undergraduate nursing students’ key demographic background is shown including sex (9 male 8.00%; 103 female 92.00%), age (35 18–20 years 31.20%; 77 > 20 years 68.80%), religiosity (51 no religion 45.50%; 61 religion 54.50%), conscious health (2 very bad 1.80%; 10 not good 8.90%; 60 normal 53.60%; 24 good 21.40%; 16 very good 14.30%), family rearing (19 single parent 17.00%; 90 both parents 80.40%; 3 grandparent(s) 2.70%), and family income (1 low 0.90%; 17 low-middle 15.20%; 80 middle 71.40%; 13 high-middle 11.60%; 1 high 0.90%) (see Table 2).

**Table 2:**
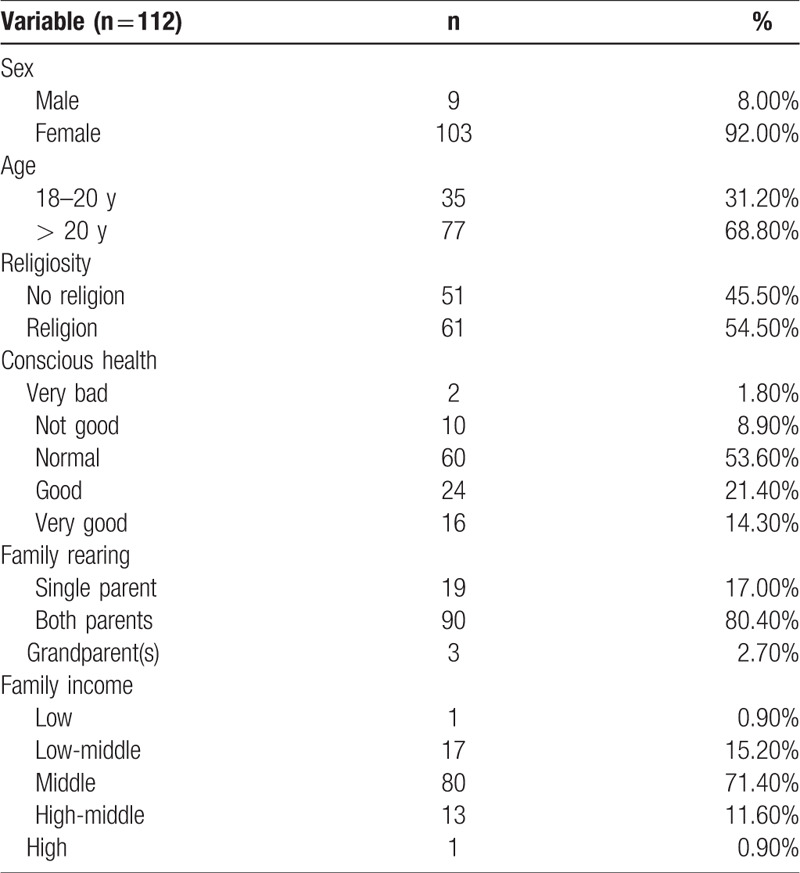
Undergraduate nursing students’ demographic background.

### 3.2 Differences between pretest and posttest: meaning of life, positive beliefs, and well-being

All undergraduate nursing students showed significant (*P* < .05; *P* < .01; *P* < .001) improvements on the meaning of life, positive beliefs, and well-being after the health promotion intervention between pretest and posttest in the study (see Table 3).

**Table 3:**
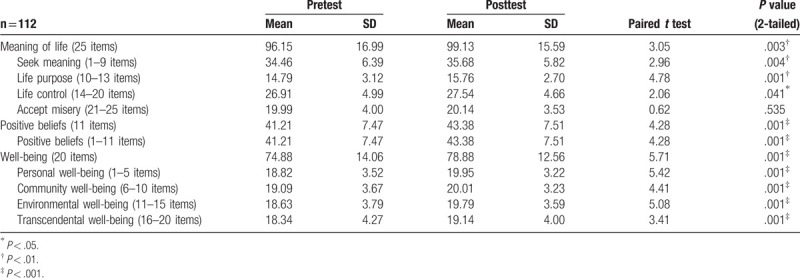
Differences between pretest and posttest: meaning of life, positive beliefs, and well-being.

### 3.3 Differences between pretest and post posttest: meaning of life, positive beliefs, and well-being

All undergraduate nursing students showed sustained significant (all *P* < .001) improvements on the meaning of life, positive beliefs, and well-being for 5 weeks after the health promotion intervention between pretest and post posttest in the study (see Table 4).

**Table 4:**
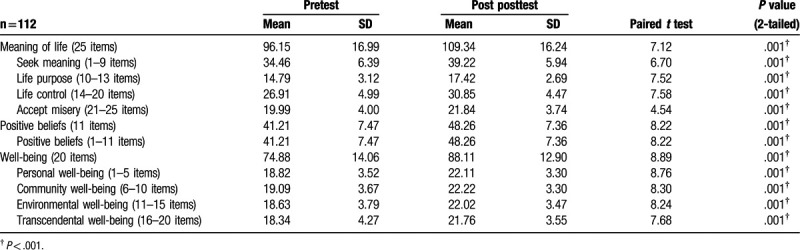
Differences between pretest and post posttest: meaning of life, positive beliefs, and well-being.

## 4 Discussion

The goal of health promotion intervention is to increase people's physical, psychological, spiritual, and social health regarding meaning of life, positive beliefs, and well-being.^[31–33]^ In this study, our research team examined 3 topics on the meaning of life, positive beliefs, and well-being and their role in a health promotion intervention with undergraduate nursing students. From this study, there were significant differences on the health promotion intervention as the same other articles.^[31–33]^ Immediately after the intervention, undergraduate nursing students displayed increased on their meaning of life, positive beliefs, and well-being, which were sustained at follow-up.

Previous literature has revealed many things: many people are deprived of food, clothing, medical care, emotional support,^[34]^ and substance use,^[35]^ which are all associated with negative health impacts. Some feel that “death is better than misery.”^[34]^ The lowest economy is associated with the highest misery index. Believing in a meaningful life, life purpose, and achieving goals is associated with decreased life misery.^[36]^ Therefore, social emotion acting is an affect control condition, and control situations to understand the emotional impairments and no longer pleasure.^[37]^ People require social support and meaning making to manage many daily life situations.^[38]^

Moreover, life control is associated with well-being, physical, psychological, spiritual, and social health promotion.^[39]^ Meaning, hope, and positive change can assist undergraduate nursing students in managing mental health problems and displaying positive attitudes in their daily lives.^[27]^ People have a strong drive to build life quality and that increases their personal and public quality of life.^[40]^ Disaster education in nursing education will increase undergraduate nursing students’ self-efficacy for managing in the psychometric properties.^[41]^ Therefore, training mental health is good for undergraduate nursing students’ self-perception, responses to situations, and perceptions of mental health.^[42]^

In addition, positive and negative beliefs are associated with the transcendental-future time perspective inventory, but are not associated with mindfulness, self-esteem, well-being, and depression.^[43]^ Individuals believe a sense of personal control and free will is associated with positive life outcomes and subjective well-being.^[44]^ Undergraduate nursing students share many ideas that are associated with mental health and well-being; therefore, they are encouraged to change their lifestyle to have well-being.^[45]^ Therefore, the environment can influence social well-being and increase one's satisfaction with personal relationships and quality of life.^[46]^

Our results have key implications for nursing educators. For example, educators should continue to use PowerPoint and e-learning platforms to promote undergraduate nursing students’ meaning of life, positive beliefs, and well-being and encourage physical, psychological, spiritual, and social health promotion. In future studies, we will continue to examine these variables and their relationship with providing health services for many patients in clinical settings or communities.

### 4.1 Limitations

This study had some limitations. First, undergraduate nursing students were limited to 112 undergraduate students who were completing the first year of a 2-year nursing program in Kaohsiung City, Taiwan, ROC; therefore, the results may not be generalizable to others.

## 5 Conclusion

Undergraduate nursing students showed both significant (pretest to posttest and pretest to post posttest) improvements on the meaning of life, positive beliefs, and well-being after the health promotion intervention; therefore, nursing educators should include these variables in the health promotion curriculum to enhance undergraduate nursing students’ physical, psychological, spiritual, and social health promotion.

## Author contributions

**Conceptualization:** Fu-Ju Tsai, Yih-Jin Hu.

**Data curation:** Fu-Ju Tsai, Yih-Jin Hu.

**Formal analysis:** Fu-Ju Tsai, Cheng-Yu Chen.

**Funding acquisition:** Fu-Ju Tsai.

**Investigation:** Fu-Ju Tsai.

**Methodology:** Fu-Ju Tsai, Yih-Jin Hu, Gwo-Liang Yeh, Cheng-Yu Chen.

**Project administration:** Fu-Ju Tsai, Yih-Jin Hu.

**Resources:** Fu-Ju Tsai, Yih-Jin Hu.

**Software:** Fu-Ju Tsai.

**Supervision:** Yih-Jin Hu, Gwo-Liang Yeh, Cheng-Yu Chen, Chie-Chien Tseng, Si-Chi Chen.

**Validation:** Yih-Jin Hu, Gwo-Liang Yeh, Cheng-Yu Chen, Chie-Chien Tseng, Si-Chi Chen.

**Visualization:** Yih-Jin Hu, Gwo-Liang Yeh, Cheng-Yu Chen, Chie-Chien Tseng, Si-Chi Chen.

**Writing – original draft:** Fu-Ju Tsai.

**Writing – review & editing:** Fu-Ju Tsai, Yih-Jin Hu.
